# Reactive Autonomous Navigation of UAVs for Dynamic Sensing Coverage of Mobile Ground Targets

**DOI:** 10.3390/s20133720

**Published:** 2020-07-03

**Authors:** Hailong Huang, Andrey V. Savkin, Xiaohui Li

**Affiliations:** School of Electrical Engineering and Telecommunications, University of New South Wales, Sydney 2052, Australia; hailong.huang@unsw.edu.au (H.H.); xiaohui.li@unsw.edu.au (X.L.)

**Keywords:** unmanned aerial vehicles, navigation, networks of drones, reactive deployment, surveillance and monitoring, sensing coverage, dynamic coverage, autonomous systems

## Abstract

This paper addresses a problem of autonomous navigation of unmanned aerial vehicles (UAVs) for the surveillance of multiple moving ground targets. The ground can be flat or uneven. A reactive real-time sliding mode control algorithm is proposed that navigates a team of communicating UAVs, equipped with ground-facing video cameras, towards moving targets to increase some measure of sensing coverage of the targets by the UAVs. Moreover, the Voronoi partitioning technique is adopted to reduce the movement range of the UAVs and decrease the revisit times of the targets. Extensive computer simulations, from the simple case with one UAV and multiple targets to the complex case with multiple UAVs and multiple targets, are conducted to demonstrate the performance of the developed autonomous navigation algorithm. The scenarios where the terrain is uneven are also considered. As shown in the simulation results, although the additional VP technique leads to some extra computation burden, the usage of the VP technique considerably reduces the target revisit time compared to the algorithm without this technique.

## 1. Introduction

Unmanned aerial vehicles (UAVs), which have been employed in various military and civilian domains [1], have become a popular tool for the surveillance of targets [2,3]. Two main reasons for the popularity of UAVs in surveillance and monitoring applications are the manufacturing cost reductions and mobility improvement due to the high-performance control of UAVs [4,5]. Considering the inefficiency of using a single large UAV, especially in large areas, it is a trend to employ multiple small UAVs to complete missions quickly [6]. For example, for the periodical area sensing mission, much time can be saved by well designing complete coverage paths for a team of UAVs that operate in a parallel manner [7,8]. Another situation is to monitor some fast changing environment such as bushfires [9], and deploying a UAV team can generally collect more temporal-spatial data than using a single UAV.

In this paper, we focus on the sensing coverage of a number of mobile targets in a region by a UAV team. This problem has several potential applications. From the sense of wireless sensor networks or Internet of Things, some sensor nodes are regarded as the targets. Consider the case where a set of sensors are deployed on the surface of the sea in the Great Barrier Reef zone to monitor the status of the coral reef system. UAVs are deployed to periodically visit the sensors to collect the sensory data [10]. From the security point, some mobile military units can be the targets of the UAVs. UAVs need to move to them to gather their dynamic status.

Different from the previous work using a sufficient number of UAVs for sensing coverage where the targets in the region of interest can be under surveillance at any time [11,12], we consider the more challenging case with a limited number of UAVs in this paper. Then, the targets cannot be covered all the time, and they need to be covered periodically. This is also more general than the case with a sufficient number of UAVs, especially when the region of interest is much larger than the sensing area of the UAVs. In this case, the revisit time among the targets should be as small as possible, as the longer time a target has not been covered, the higher the uncertainty level will be with the target. The problem of interest is how to organize the motions of the UAVs to accommodate the movements of the targets. Different from many existing approaches that construct the path first and design tracking algorithms, we jointly consider the motion control and the trajectory generation. We start from a simple scenario with a single UAV. We develop a reactive real-time sliding mode control algorithm, which navigates the UAV towards the pursuit target (PT). Here, the PT accounts for not only the distance from the UAV to the target but also the time duration this target has not been under coverage. In the meanwhile, the UAV’s dynamics is taken into account. Therefore, this navigation algorithm can control the motion of the UAV and plan the path simultaneously. We then move onto the more complex case with multiple UAVs. Beyond the motion control and path planning, the resource allocation issue rises. Then, there needs a coordination scheme among the UAVs. To cooperate, the UAVs share the measured information with others via wireless communication. Then, all the UAVs follow a designed PT selection rule, so that any PT can only be selected once at any time. Whenever two UAVs are close enough, one of them stops pursuing the selected target and takes actions to avoid collisions. Moreover, we adopt the Voronoi partition (VP) technique to reduce the movement range of each UAV. To evaluate the proposed method, we conduct extensive computer simulations. We start from the simple case with one UAV and multiple targets, and then move onto the complex case with multiple UAVs and multiple targets. The scenarios where the terrain is uneven are also considered. As shown in the simulation results, having more UAVs significantly increases the frequency of surveying the targets. Furthermore, although the additional VP technique leads to some extra computation burden, the usage of the VP technique considerably reduces the target revisit time compared to the algorithm without this technique. Moreover, the UAV linear speed has an important impact on the target revisit time, while the influence of the angular speed is small.

The main contribution is the developed autonomous navigation algorithm that can guide a group of UAVs to reactively monitor a set of moving targets. This algorithm is computationally efficient and can be implemented easily in real time. Extensive computer simulations are presented to show the effectiveness of the proposed method.

The remainder of the paper is organized as follows. Section 2 discusses the related work. Section 3 first presents the dynamics of the UAVs and sensing coverage model. Then, the problem under consideration is stated. Section 4 presents the proposed UAV navigation approaches based on a sliding mode control algorithm. Illustrative examples and computer simulations are given in Section 5 to demonstrate the effectiveness of the proposed approaches. Finally, Section 6 presents a brief conclusion.

## 2. Related Work

The problem of target surveillance is not a new problem, and it has been considered from different levels. For the low level of sensing, many data fusion methods have been developed to integrate the observations from sensors to obtain a better estimation of the target. Approaches based on vision sensors have attracted much attention in recent years. The authors of [13,14] propose methods to detect and localize a target using images taken by a UAV. The authors of [15,16] further propose fast path planning schemes for a UAV to track a target based on estimated location and distance between the target and the UAV. Beyond using a single UAV, researchers have carried out investigations on using multiple UAVs to cover a single target [17,18,19]. The deployment of multiple UAVs enables the observation of the target from different directions and also reduces the probability of losing the target especially in urban areas where occlusion often exists due to the tall buildings.

In the aforementioned approaches, the focus is on the quality of the target detection. When multiple targets are to be monitored, another problem rises, i.e., how to allocate the UAV resource. The resource allocation significantly affects the quality and efficiency of sensing coverage. The stationary target surveillance has been studied in varying applications, and the targets can be military units, buildings and sensor nodes. Many existing approaches have been employed to address the path planning issue in these applications. When there is only one UAV, the problem can be formulated as the conventional travelling salesman problem (TSP) with the objective of minimizing the time to complete the survey of all the stationary targets [20]. Moreover, some variants of TSP have also been used to address the path planning problem such as TSP with neighbourhoods [21]. Specifically, each target can be surveyed from a certain distance. For example, a UAV can collect the sensory data from a sensor node from a distance. Then, the constructed path does not necessarily pass the targets. Thus, the path length can be shortened. When multiple UAVs are available and the targets spread in the field, the vehicle routing problem (VRP) is a common tool to formulate the path planning problem [22]. In the case of having enough UAVs, an interesting problem is where to deploy them so that an optimal sensing coverage of the targets can be achieved [11,12]. When the targets move in the considered region, some reactive algorithms are needed to maintain the sensing quality [2,23].

The sensing coverage of moving targets can be regarded as a generalization of TSP in the single UAV case. However, the approaches to the original TSP cannot be used to generate the path for the UAV because following a pre-computed path cannot guarantee the successful visitation of all the moving targets. Then, the path has to be recomputed once the targets move. Some general optimization algorithms, such as genetic algorithms [24], have been exploited to reconstruct the path online. It is easy to understand that executing this kind of optimization algorithms requires excellent computing ability. The work in [25] considers the problem of surveying multiple targets by multiple UAVs. The authors assume that the UAVs move much faster than the targets so that when a UAV arrives at a target’s last measured location, the target can be covered by the UAV. Thus, their approach still addresses the stationary case with the movements of the targets viewed as perturbations. The work in [26] investigates the problem of covering a group of moving targets using the minimal number of UAVs. The authors focus on two problems: (1) covering all the targets for the entire duration of mission and (2) covering each target at least once during the duration of mission, and they use the generalized version of the minimum flow problem to address these problems. The authors of [27] present a strategy for a one-off mission planning for a UAV team, where *n* UAVs are required to move to *n* destinations, e.g., to cover some targets are the destinations. Moreover, A* was adopted to construct the path from a UAV to any target destination, and then the optimal combination of paths is figured out by evaluating each possible combination.

In this paper, the VP technique is adopted. This technique is widely used to partition a plane into regions close to each of a given set of objects. Different domains using VP and its variants include, but not limited to sensor coverage, area search with multiple robots and complete area coverage using multiple robots. The authors of [28] propose a VP-based method to determine the optimal deployment of a set of UAV base stations to serve ground cellular users. The authors of [29] use this technique to find the minimum number of charging stations and their locations for drone parcel delivery. Different from these references, the current paper adopts this technique to group a set of moving targets, in order to improve reduce the target revisit time, which is equivalent to improving the sensing coverage quality.

## 3. System Model and Problem Statement

Consider a surveillance system consisting of *N* UAVs la belled i=1,2,…,N. The UAVs fly in a plane that is parallel to the ground. The altitude of this plane is *Z*, and it is within the allowed deployment range. Let $p_{i}\left(t\right)\in R^{2}$ denote the location of the projection of UAV *i* (i=1,…,N) on the ground at time *t* and $h_{i}\left(t\right)\in R^{2}$ ($∥h_{i}\left(t\right)∥=1$) denote the heading of the UAV. The motion of UAV *i* is described by

(1) $${\dot{p}}_{i}\left(t\right)=v_{i}\left(t\right)h_{i}\left(t\right),$$

(2) $${\dot{h}}_{i}\left(t\right)=u_{i}\left(t\right)$$

where $v_{i}\left(t\right)\in R$ is the linear speed and $u_{i}\left(t\right)\in R^{2}$ is to adjust the UAV’s heading. Both $v_{i}\left(t\right)$ and $u_{i}\left(t\right)$ are the control inputs. The following constraints hold,

(3) $$∥u_{i}\left(t\right)∥\leq U_{max},\;\;\;v_{i}\left(t\right)\in \left[-V_{max},V_{max}\right],$$

(4) $$\left(h_{i}\left(t\right),u_{i}\left(t\right)\right)=0$$

for all *t*. Here, ∥·∥ denotes the standard Euclidean vector norm, $\left(\cdot ,\cdot \right)$ denotes the scalar product of two vectors, and $U_{max}$ and $V_{max}$ are the given constants. The condition (4) guarantees that the vectors $h_{i}\left(t\right)$ and $u_{i}\left(t\right)$ are always orthogonal. The kinematics of many UAVs can be described by non-holonomic models (1)–(4), see, e.g., in [30] and the references therein (Note that in this dynamic model, the linear speed of a UAV can be negative. This differs from many other publications where the linear speed can only take positive values; see [31,32,33]. The advantage is that when the UAV needs to turn the moving direction for more than 90 degrees, it does not need to make a circle with the maximum angular velocity. Instead it can apply a negative speed.). The main notations used throughout the paper and their meanings are listed in Table 1.

**Figure 1:**
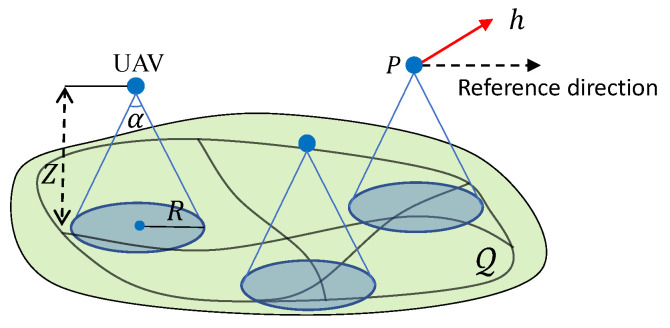
Illustration of the surveillance system.

The goal of this system is to survey targets moving on the ground. Thus, each UAV is equipped with a ground-facing camera that has a given observation angle α∈(0,π); see Figure 1. Each UAV, say *i*, can see a circular area on the ground centred at $p_{i}\left(t\right)$ with the radius of

(5) $$R:=Z\tan\left(\frac{\alpha }{2}\right).$$

It is clear that in (5), the altitude *Z* and the angle α determine the vision area. As mentioned above, the altitude *Z* must be within an allowed range which is regulated by the local government for the safety of other flying objects or some infrastructure such as power lines. The angle α is determined by the camera in use, and different types of cameras result in varying values of α.

There is a set of *M* targets to be surveyed moving in a given region $Q\subset R^{2}$ on the ground. Throughout this paper, the terms of survey, cover and visit are used interchangeably. Let $q_{j}\left(t\right)\in Q$ denote the Cartesian coordinates of target *j* (j=1,…,M) on the ground at time *t*. Each target moves on a pre-defined trajectory with an approximately constant speed along its trajectory. This assumption holds in some practical scenarios. For example, a car usually drives with a roughly constant speed on the highway. Even this assumption does not hold, some more advanced estimation and prediction algorithms can be used to estimate and predict the targets’ locations. We also assume that when a target is under coverage by a UAV, its location and speed can be measured by the UAV via some image processing techniques. When it is not under coverage by any UAVs, the measured location and speed are used to predict its current location, which is denoted by ${\hat{q}}_{j}\left(t\right)$. Let ϵ denote the maximum error of the measured speed of the targets and δ denote the sampling time. Then, the single-step prediction error of a target’s location is upper bounded by δϵ.

When N≥M, each target can be monitored by a UAV at any time. When N<M, there will be some time during which a target is not under coverage (Note that we consider the case where the *M* targets are spread in Q. Having multiple targets under coverage by one UAV is an abnormal situation, and we assume that if there is such a situation, it will not last long.). The longer time a target has not been under coverage, the higher the uncertainty level is with this target. Thus, when the resource is limited, an important feature of the surveillance system is to maintain the uncertainty of the targets at the lowest level. This can be stated as an optimization problem that minimizes the maximum revisit time among the targets. Let $\tau_{j}$ denote the time gap between two consecutive visits of target *j*, which depends on the motion of the UAVs, and let τ denote the maximum of such time gap among the targets, i.e.,

(6) $$\tau :=\max_{j=1,\ldots ,M}\tau_{j}.$$

With the single-step location prediction error bound δϵ, the maximum location prediction error for a time period τ is bounded as follows,

(7) $$\max_{j=1,\ldots ,M}∥q_{j}\left(t\right)-{\hat{q}}_{j}\left(t\right)∥\leq \delta \epsilon \frac{\tau }{\delta }=\epsilon \tau .$$

Moreover, to guarantee that the surveillance system can accommodate this maximum prediction error, the following condition should be satisfied,

(8) ϵτ≤R.

Otherwise, when a UAV flies to the predicted location of a target, the UAV cannot have the target in view. In other words, this target is lost. To survey this target, the UAV needs to take some extra operations to search the target.

Problem Statement: for the given constants *Z*, α, ϵ, $U_{max}$, and $V_{max}$, we aim at navigating *N* UAVs to survey *M* moving targets in Q, so that all the targets are under coverage periodically.

## 4. Methodology and Main Results

The question to be answered is how to control the motion of a UAV team so that the UAVs can periodically survey the moving targets.

### 4.1. Methodology

**Figure 2:**
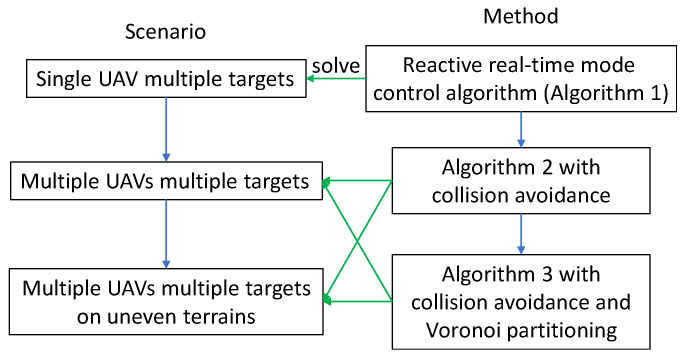
Methodology.

In this section, we address the considered problem following the below methodology, which is shown in Figure 2.

We start from a simple case with one UAV to discuss how to control the motion of the UAV and analyze the number of targets it can survey (Section 4.2). A reactive real-time sliding mode control algorithm is proposed that navigates the UAV to periodically survey a group of targets. As the targets are mobile, following a predefined trajectory does not guarantee the successful visit of all the targets. Thus, we adopt propose a metric to characterize which target should be surveyed next. This metric combines the distance information between the UAV and a target and how long the target has not been surveyed. The target with the highest value of this metric is regarded as the pursuit target (PT). At any time, the UAV moves towards the PT.

With this algorithm, we furthermore address the more complex case with multiple UAVs. As the collision issue arises when multiple UAVs operate simultaneously, the UAVs survey targets with a coordinating scheme to avoid collision. In Section 4.3, the algorithm to navigate a UAV team is presented, and in Section 4.4, the algorithm is improved by adding the Voronoi partitioning technique to reduce the movement range of the UAVs, so that the target revisit time can be shortened. Finally, we present the extension of the proposed method to the cases with targets move on uneven terrains (Section 4.5).

### 4.2. Navigation Algorithm for a Single UAV

In this subsection, we consider the case of using a single UAV to periodically cover a set of moving targets. Different from many existing methods which determines the high-level visiting order of the targets for the UAV without accounting for the low-level control of the UAV, we consider the visiting target selection and the motion control of the UAV simultaneously. We first present a sliding mode algorithm to control the motion of the UAV.

**Figure 3:**
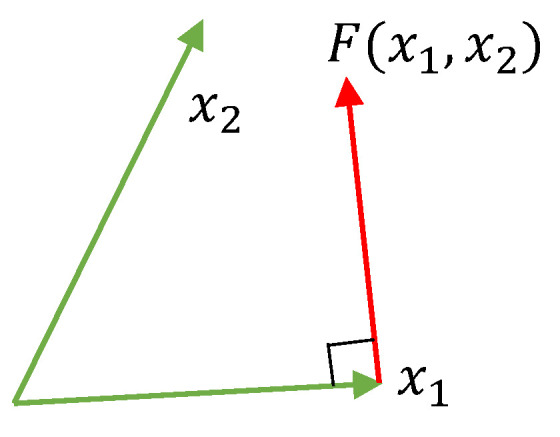
Illustration of the function $F(x_{1},x_{2})$.

Let $x_{1},x_{2}\in R^{2}$ be two non-zero vectors, and $∥x_{1}∥=1$. Introduce the function F(·,·) mapping from $R^{2}\times R^{2}$ to $R^{2}$ as

(9) $$F\left(x_{1},x_{2}\right):=\begin{array}{c} \left\{\begin{array}{c} 0,\;\;\;\;\;\;\;\;\;\;\;\;\;f(x_{1},x_{2})=0,\\ \frac{f(x_{1},x_{2})}{∥f(x_{1},x_{2})∥},\;\;\;f\left(x_{1},x_{2}\right)\neq 0, \end{array}\right. \end{array}$$

where $f\left(x_{1},x_{2}\right):=x_{2}-\left(x_{1},x_{2}\right)x_{1}$. The rule (9) is defined in the plane of vectors $x_{1}$ and $x_{2}$, and the vector $F(x_{1},x_{2})$ is orthogonal to $x_{1}$ and directed "towards" $x_{2}$, see Figure 3. It is also clear that $F(x_{1},x_{2})=0$ if $x_{1}$ and $x_{2}$ are co-linear, and $∥F(x_{1},x_{2})∥=1$ otherwise. Moreover, introduce the function $g(x_{1},x_{2})$ as follows,

(10) $$g\left(x_{1},x_{2}\right):=\begin{array}{c} \left\{\begin{array}{c} 1,\;\;\;\;\;(x_{1},x_{2})>0,\\ -1,\;\;\;(x_{1},x_{2})\leq 0. \end{array}\right. \end{array}$$

Let a(t) be a vector pointing from the current location of the UAV p(t) to the predicted location of a target $\hat{q}\left(t\right)$, i.e., $a\left(t\right):=\hat{q}\left(t\right)-p\left(t\right)$. We use the following sliding mode guidance law to control the motion of the UAV,

(11) $$u\left(t\right)=U_{max}g\left(h\left(t\right),a\left(t\right)\right)F\left(h\left(t\right),a\left(t\right)\right),$$

(12) $$v\left(t\right)=V_{max}g\left(h\left(t\right),a\left(t\right)\right).$$

**Remark** **1.** *The navigation law (11) adjusts the heading of the UAV towards the predicted target location with the maximum angular speed, and the law (12) computes the linear speed of the UAV. Note that the linear speed obtained from (12) can be negative, as the angle between the vectors h(t) representing the motion direction of the target and a(t) representing the desired motion direction can be larger than $\frac{\pi }{2}$. The advantage of this algorithm is that the UAV does not need to make a circle to turn back. In other words, the UAV can quickly adjust its motion direction towards the target.*

We are now in a position to use the aforementioned motion control algorithm to deal with the task of surveying multiple moving targets. As the targets can move, following a predefined path does not ensure that all the targets can be surveyed by the UAV. Thus, it is necessary to develop an online trajectory planning method to accommodate the targets’ movements. When the targets are stationary, the problem of interest can be converted to the conventional TSP for the single UAV case. TSP aims to find the trajectory for the UAV so that it takes the minimum time to visit all the targets. There are many existing solutions, including exact algorithms and heuristic algorithms. Among them, the exact algorithms take too long to solve even a medium-scale problem. There are some heuristic algorithms, such as the nearest neighbor (NN) algorithm, that can quickly solve the problem as they trade the optimality for speed. The proposed method shares the fundamental idea of the NN algorithm. Differently, the term of NN in this paper depends on not only the distance, but also on the uncertainty level of a target.

The uncertainty level of a target depends on the time the target has not been under coverage since the last visit [34] (In the case of using sensor nodes to monitor the status of the coral reef system in the Great Barrier Reef zone, the longer time a sensor node has not been visited, the more data it stores. If a node is not visited for a long time, the stored data may even overflow.). In this paper, we assume that if a target was visited at time $t_{1}$, the uncertainty level maintains a very low level for a period of time *T* (T≥0). After that, it increases with time. Let β(t) be such a function for all $t\in [t_{1},t_{2})$. Here, $t_{2}$ is the time at which the target is visited for the next time, which is unknown in advance. The function β(t) satisfies the following conditions,

(13) $$\begin{array}{cc} \; & \beta \left(t\right)=0,\;\;\;t\in \left[t_{1},t_{1}+T\right],\\ \; & \beta \left(t\right)>0,\dot{\beta }\left(t\right)>0,\;\;\;t\in \left(t_{1}+T,t_{2}\right). \end{array}$$

**Figure 4:**
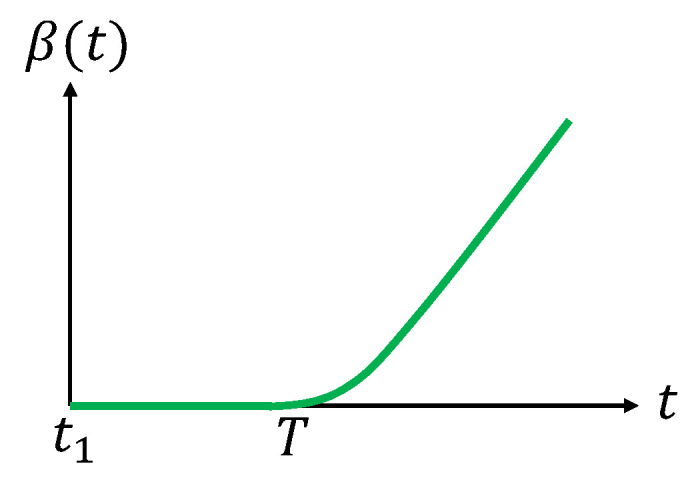
Illustration of the function β(t).

When the target is visited by a UAV at time $t_{2}$, the uncertainty level of this target drops to 0, maintains 0 for a period of *T*, and then increases with time again; see Figure 4. An example of the function β(t) satisfying (13) is as follows,

(14) $$\beta \left(t\right)=\begin{array}{c} \left\{\begin{array}{c} 0,\;\;\;\;\;\;\;\;\;\;\;\;\;t\in [t_{1},t_{1}+T]\\ t-t_{1}-T,\;\;\;t\in \left(t_{1}+T,t_{2}\right). \end{array}\right. \end{array}$$

In our proposed solution, we use the notation $\beta_{j}\left(t\right)$ to describe the uncertainty level of target *j* at time *t*. Introduce a new metric $\gamma_{j}\left(t\right)$ for target *j* at time *t*:

(15) $$\gamma_{j}\left(t\right):=\frac{\beta_{j}\left(t\right)}{d_{j}\left(t\right)},$$

where $d_{j}\left(t\right)$ is the distance between the UAV and the target *j* on the horizontal plane at time *t*. This metric couples the information of distance and uncertainty level. The target with the maximum value of γ(t) is named as the pursuit target (PT).

The proposed method to reactively navigate a single UAV to survey a set of moving targets is summarized in Algorithm 1. It consists of three main steps: First, the UAV chooses the PT. Then, the UAV computes the control inputs u(t) and v(t) according to (11) and (12), and applies them to make a movement for a sampling time δ. During the movement, the UAV updates the status of the targets that have been in its view. The UAV will choose the next PT when the current PT is covered. It is clear that the UAV can reactively move towards the PT to accommodate the movement of the PT. Therefore, we call this algorithm the online reactive navigation algorithm.
**Algorithm 1:** Online reactive navigation algorithm for a single UAV.
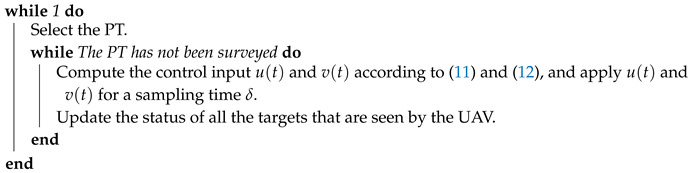

**Remark** **2.** *The proposed online reactive navigation algorithm, i.e., Algorithm 1, is easy to implement on a UAV in real-time. From Algorithm 1 we can see that the UAV only needs to compute two control inputs: u(t) and v(t) according to (11) and (12). Obviously, these simple calculations are computational efficient. Whenever the UAV sees a target, the on-board sensors take the measurements of the target.*

When there is only one UAV, the condition (8) is not guaranteed to hold. In this case, an interesting issue is how many targets in Q can one UAV survey subject to condition (8). Such a number depends on how the targets spread in Q. When they are relatively close, this number is larger, while when they are relatively far from each other, it is smaller. Clearly, in the former case, the number of targets can be infinite. It is more interesting to analyze the upper bound on the number of targets in the latter case.

Let *L* be the length of the shortest path that connects two farthest points in Q. Moreover, the curvature at any point on this path is no larger than $\frac{U_{max}}{V_{max}}$. Then, this path can be followed by a UAV modeled by (1)–(4). Let $V_{T}$ be the maximum speed of targets. We assume that the UAV can move faster than the targets, i.e., $V_{T}<V_{max}$. With *M* targets in Q, no matter how they move, the length of the UAV trajectory is bounded by ML. To complete the longest trajectory within the time τ, we have the following inequality:

(16) $$ML\leq V_{max}\tau .$$

Together with (8), we can obtain the upper bound on *M*:

(17) $$M\leq \frac{RV_{max}}{\epsilon L}.$$

**Remark** **3.** *This analysis shows that when M satisfies (17), these targets can be covered by the UAV. It is also worth pointing out that (17) is a conservative bound. It considers the worst case where any pair of the targets are L away from each other. However, in practice, such a case only exists when M=2, where the two targets are at the two ends of the path having the length of L. The UAV flies back and forth, and the total length of its trajectory is 2L. When M>2, the total length of the UAV’s trajectory is less than ML. Then, when the UAV travels for ML, more targets can be surveyed. We will show this in Section 5.*

### 4.3. Navigation Algorithm for Multiple UAVs

When multiple UAVs are available, the target revisit time can be significantly reduced. The navigation of each UAV among the team is still based on Algorithm 1, but there should be some coordinating schemes so that the surveying mission can be carried out efficiently by the UAVs. The navigation algorithm for each UAV among the team is summarized in Algorithm 2, and the differences with Algorithm 1 are explained below.
**Algorithm 2:** Online reactive navigation algorithm for each UAV of the team.
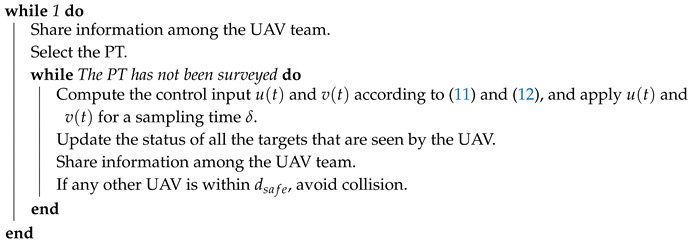


**Data exchange:** When a target is within the vision cone of a UAV, the target status, such as the ID, speed, and location, can be collected by the UAV. As the targets can move, it is possible for some target which was closer to one UAV to be closer to another UAV in the future. Thus, the UAVs share their own locations and the information about the targets with each other.

**PT selection:** Different from the single UAV case, the selection of PT for one UAV, say UAV *i*, needs to take into account not only the uncertainty levels and estimated locations of the targets and the location of UAV *i* but also the locations of other UAVs, which can be obtained by the data exchange process. The coordinating rule is stated as follows. Every UAV computes a *N*-by-*M* matrix Γ with $\gamma_{ij}\left(t\right)=\frac{\beta_{j}\left(t\right)}{d_{ij}\left(t\right)}$ as elements, where $d_{ij}\left(t\right)$ is the distance between UAV *i* and target *j* on the horizontal plane at time *t*. To select the PT, UAV *i* finds the element in the *i*th row of the matrix Γ, say $\gamma_{ij}\left(t\right)$, such that $\gamma_{ij}\left(t\right)$ is the largest element in the *j*th column. In the case where there is another element in the *j*th column having the same value as $\gamma_{ij}\left(t\right)$, target *j* is assigned as the PT of the UAV with the smaller index. This case occurs when two UAVs are at the same distance away from target *j*. Clearly, this coordinating scheme will assign each UAV a unique PT at any time, since N<M.

**Collision avoidance:** Although different UAVs have different PTs, it is still possible that one UAV collides with others during the movement, as the targets can move. There is a great amount of research results on the topic of collision avoidance of mobile robots [35,36]. In this paper, we consider a simple approach. To avoid collision, all the UAVs obey the following rule: whenever two UAVs are within a safe distance $d_{safe}$, the UAV with the smaller index continues to move towards its PT, while the other UAV takes actions to avoid collision. For example, it can hover at its current position or move away from the former UAV. Thus, it is required that the collision avoidance ability is embedded, which is common in the commercial UAVs. When they are at least $d_{safe}$ away from each other, the latter re-starts to move towards its PT. Clearly, as each UAV has a unique index and a UAV knows the indices of the nearby UAVs via the data exchange, this simple approach can effectively avoid collisions between UAVs.

We now consider the number of UAVs required to survey *M* targets moving in Q. When there are *N* UAVs, the total length of their trajectories is upper bounded by ML+NL. This can be obtained by breaking the trajectory for one UAV (whose length is ML as mentioned in Section 4.2) and adding *N* extra subpaths with the length of *L*. As these trajectories are followed by *N* UAVs, the following inequality holds,

(18) $$ML+NL\leq NV_{max}\tau .$$

Together with (8), we can obtain the lower bound on *N*:

(19) $$N\geq \frac{M\epsilon L}{RV_{max}-\epsilon L}.$$

**Remark** **4.** *Similar to the single UAV case, (19) is also a conservative bound.*

### 4.4. VP-Based Navigation Algorithm for Multiple UAVs

In Section 4.3, we present an algorithm to navigate multiple UAVs. Via computer simulations, we have observed one drawback of the proposed algorithm. Specifically, each UAV may traverse the major area of the region Q. This may result in a long revisit time of each target. To avoid this drawback and reduce the target revisit time, in this subsection, we present a Voronoi partition (VP) based navigation algorithm for multiple UAVs.

One of the most important techniques of this VP-based navigation algorithm is the partition of the region according to the locations of the targets and the UAVs. In particular, a target falls into the Voronoi cell of a UAV if their distance is no larger than the distance between this target and any other UAV. Formally, let $C_{i}\left(t\right)$ denote the Voronoi cell of UAV *i*. Target *j* belongs to $C_{i}\left(t\right)$ if the following condition holds, (Note that when the equality of (20) holds, the target *j* is assigned to the Voronoi cell with the smaller index.)

(20) $$d_{ij}\left(t\right)\leq d_{hj}\left(t\right),\forall h=1,\ldots ,N,\;\;h\neq i.$$

As mentioned in Section 4.3, the UAVs share the information about the targets and their own locations. With this information, each UAV knows which target is closer to itself than any other UAVs, so that it can construct its Voronoi cell. Then, each target chooses its PT from its own Voronoi cell. Such a VP-based method is summarized in Algorithm 3.

It is easy to understand that the partition of the targets according to (20) assigns each target to a unique Voronoi cell. Thus, at any time, each UAV has its unique PT. Algorithm 3 has one more operation that needs to be executed by each UAV than Algorithm 2, i.e., the determination of the Voronoi cell. For each target, each UAV needs to compute the distance from this target to *N* UAVs. Then, for *M* targets, the additional computation complexity is O(MN). As will be shown in the section of simulation results, such an additional computation results in a large percentage of revisit time reduction.
**Algorithm 3:** VP-based Online reactive navigation algorithm for each UAV of the team.
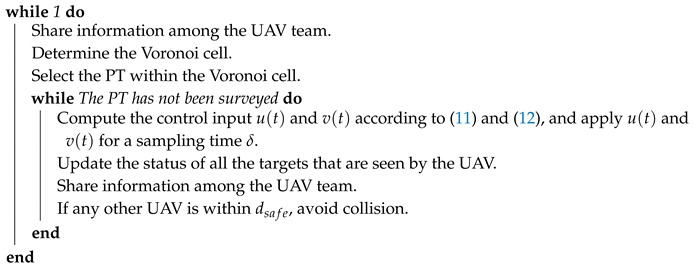


### 4.5. Extension to Uneven Terrains

So far, we have presented UAV navigation algorithms that enable a UAV team to periodically cover a set of targets that move on a flat ground. In this subsection, we point out that the proposed approaches can also be used in the cases with uneven terrains under some conditions.

Different from the flat ground situation, the notation Q now represents a region on an uneven terrain. Let $Z_{max}$ be the altitude of the highest point in Q. For the safety purpose, it is necessary deploy the UAVs at an altitude higher than $Z_{max}$:

(21) $$Z\geq Z_{max}+Z_{safe},$$

where $Z_{safe}$ denotes the minimum vertical distance the UAVs should be away from the terrain.

Moreover, as the region Q is now uneven, any target in this region should be with a 3D coordinates rather than the 2D coordinates in the flat ground case. Let $\alpha_{0}$ denote the largest elevation angle of the region Q. We consider the following condition,

(22) $$\alpha_{0}<\frac{\pi -\alpha }{2}.$$

**Figure 5:**
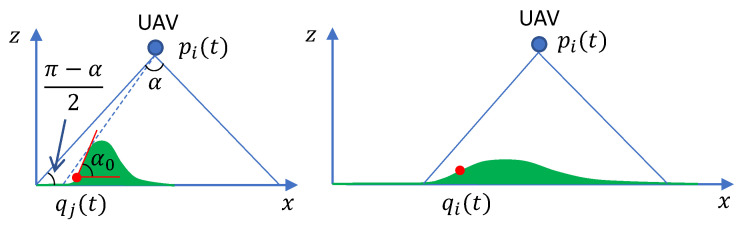
Vision cone with angle α. On the left side, the maximum elevation angle $\alpha_{0}$ is larger than $\frac{\pi -\alpha }{2}$. Thus, some part of the terrain inside the vision cone of the UAV is occluded. The right hand side shows that when $\alpha_{0}<\frac{\pi -\alpha }{2}$ holds, the terrain inside the vision cone can be seen by the UAV.

The motivation behind this condition is to avoid occlusion because of the terrain. An illustration is shown in Figure 5. On the left hand side of Figure 5, the above condition is not satisfied. Then, the target at the position $q_{j}\left(t\right)$ cannot be covered by the UAV at $p_{i}\left(t\right)$. When this condition holds, the part of Q that is inside the vision cone of a UAV can be covered by the UAV without any occlusion; see the right hand side of Figure 5. Then, when the condition (8) is satisfied, the UAV can cover the target when the UAV reaches the estimated location of the target $\hat{q}\left(t\right)$. Readers are referred to our previous work [11] on the topic of static surveillance accounting for occlusion.

Therefore, when the region Q on the uneven terrain satisfies conditions (21) and (22), the approaches presented above can still be used. It is also worth pointing out that in practice, the condition (21) is usually easy to be satisfied, while the condition (22) may not be satisfied for some quite uneven terrain like the one on the left hand side of Figure 5. When the condition (22) cannot be satisfied, an extra searching operation can be conducted when the UAV reaches the prediction location of the target. The target can be found after the UAV searches the unseen area.

## 5. Simulations

**Figure 6:**
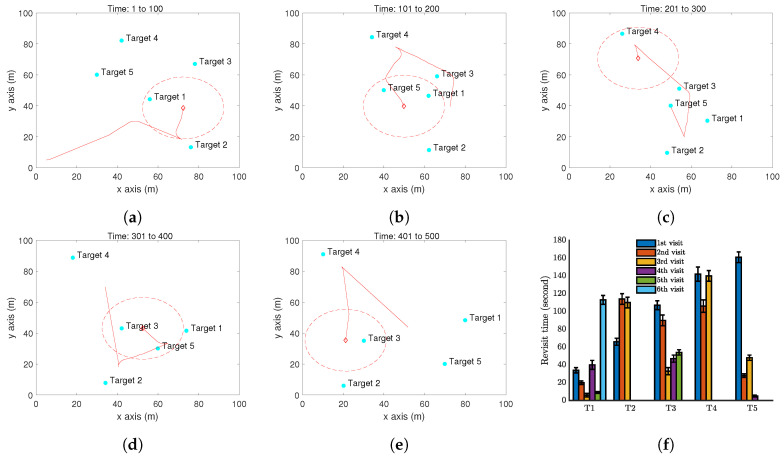
Simulation results with a single UAV and five moving targets on a flat ground by Algorithm 1. (**a**–**e**) The trajectory of the UAV (red curve) and the positions of targets (cyan dots) at different time instants. The red dash circle is the boundary of the UAV vision cone (https://youtu.be/jLyacSXMZl4). (**f**) Revisit time of each target.

**Figure 7:**
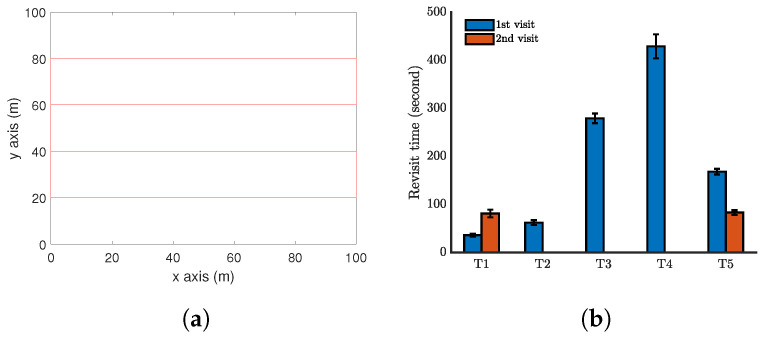
Simulation results when the UAV follows a complete coverage path. (**a**) The UAV path. (**b**) Revisit time of each target.

The performance of the proposed approach is demonstrated by computer simulations carried out with Matlab. We first present one simulation with a single UAV periodically monitoring five moving targets in a square zone (100 m by 100 m) in Figure 6. Here, Z=20 m, $\alpha =\frac{\pi }{2}$, ϵ=0.1 m/s, $V_{max}=1$ m/s, and $U_{max}$ = 0.2. The function β(t) is set as the example (14) with T=10 s (It is worth pointing out that any other functions of β(t) can also be used. Also, the parameters used are only for computer simulations.). The simulate time for this case is 500 seconds. The trajectory of the UAV is shown in Figure 6a–e. A video recording the movements of the targets and the UAV in the simulation period is available at: https://youtu.be/jLyacSXMZl4. Figure 6f shows the distribution of the revisit time of each target in 10 independent simulations, in order to capture the uncertainty in the target location prediction. Target 3 has been surveyed six times, Target 1 and Target 5 have been surveyed five times, and Target 2 and Target 4 have been surveyed three times in 500 seconds. The maximum revisit time among these targets is about 160 seconds. To further demonstrate the performance of the proposed method, a comparison is made with a benchmark method. As we have not seen any existing work focusing on the currently considered problem, we regard a complete coverage path method as the benchmark. The complete coverage path method aims at generating a path that can completely cover the area of interest. This method is widely used in agriculture, search and rescue, exploration, etc. For the above considered setting, a complete coverage path is generated for the UAV; see Figure 7a. Note that this path is generated by straight lines connecting some waypoints. The UAV cannot exactly follow this path. Specifically, at the waypoints, the UAV has to make a smallest circle to turn the moving direction, and this is not shown in Figure 7a. All the targets move in the same way as above, and the target revisit time is shown in Figure 7b. Clearly, following the complete coverage path results in a much larger revisit time than the proposed method. The reason is that following the complete coverage path makes the UAV passive survey the targets.

**Figure 8:**
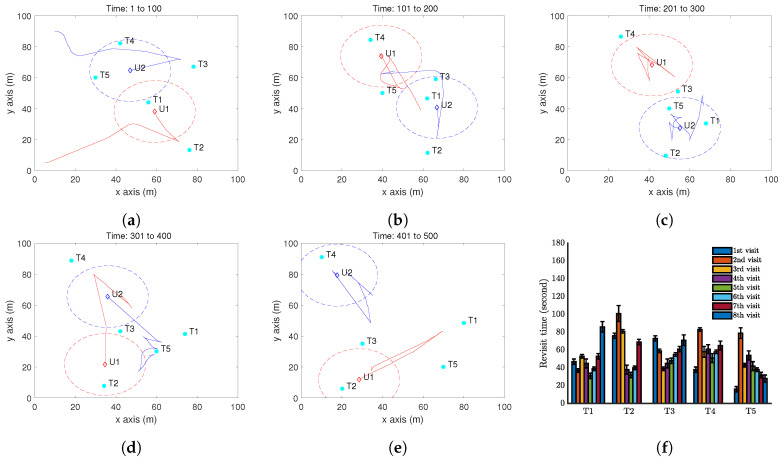
Simulation results with two UAV and five moving targets on a flat ground by Algorithm 2. (**a**–**e**) show the trajectory of the UAV (red curve) and the positions of targets (cyan dots) at different time instants. The red dash circle is the boundary of the UAV vision cone (https://youtu.be/h46R4QvPkQs). (**f**) Revisit time of each target.

Now, we present a case with two UAVs to test Algorithm 2. We first consider case with one more step forward from the above case. In other words, all the settings remain the same except that two UAVs are deployed. The results are shown in Figure 8. Comparing with Figure 6f, we can clearly see the advantage of introducing one more UAV: the target revisit is reduced to about 100 seconds.

**Figure 9:**
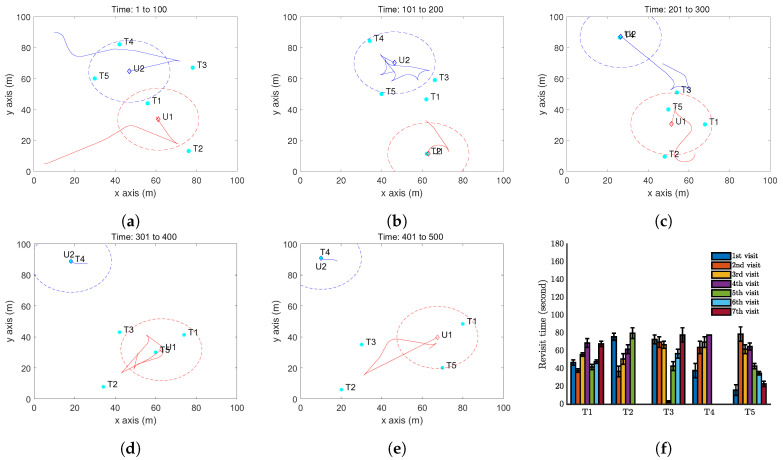
Simulation results with two UAV and five moving targets on a flat ground by Algorithm 3. (**a**–**e**) The trajectory of the UAV (red curve) and the positions of targets (cyan dots) at different time instants. The red dash circle is the boundary of the UAV vision cone (https://youtu.be/8wIfqLDeqHE). (**f**) Revisit time of each target.

Moreover, we present a case with two UAVs to test Algorithm 3. The results are shown in Figure 9. Comparing Figure 6f, Figure 8f and Figure 9f, we can clearly see the advantage of the VP technique: the target revisit is further reduced to about 80 seconds.

**Figure 10:**
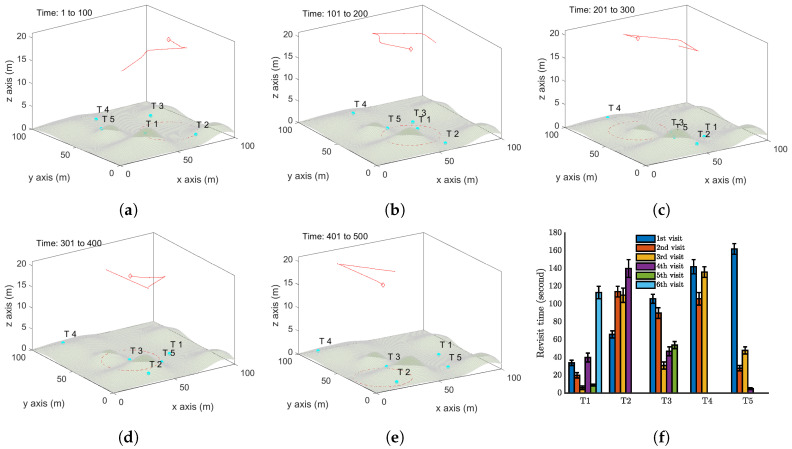
Simulation results with a single UAV and five moving targets on an uneven terrain by Algorithm 1. (**a**–**e**) The trajectory of the UAV (red curve) and the positions of targets (cyan dots) at different time instants. The red dash circle is the boundary of the UAV vision cone. (**f**) Revisit time of each target

**Figure 11:**
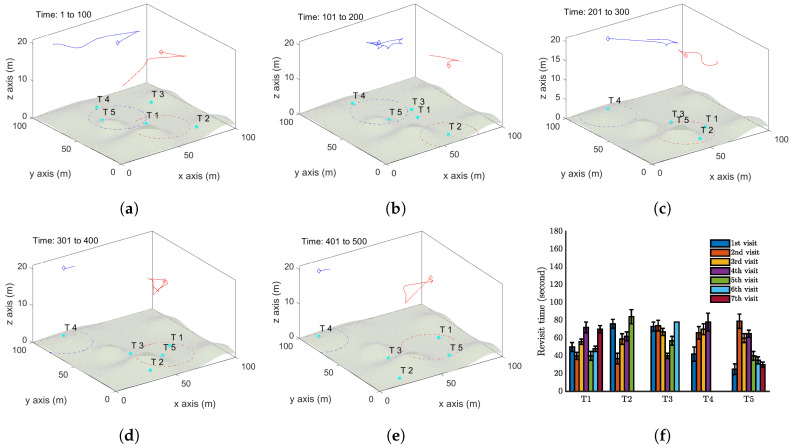
Simulation results with two UAVs and five moving targets on an uneven terrain by Algorithm 3. (**a**–**e**) show the trajectories of the UAVs. (**f**) Revisit time of each target.

We now demonstrate some simulation results where the targets move on an uneven terrain. We first present a single UAV case with five moving targets in Figure 10. The movements of these targets are similar to those in the first case, see Figure 6. The only difference lies in the uneven terrain. All the system parameters remain the same as the first case. The trajectory of the UAV is shown in Figure 10a–e, and Figure 10f displays the revisit times of the targets. Comparing with Figure 6f, Target 5 has the largest revisit time, which is around 165 seconds. Figure 11 reports the case with two UAVs by applying Algorithm 3.

**Figure 12:**
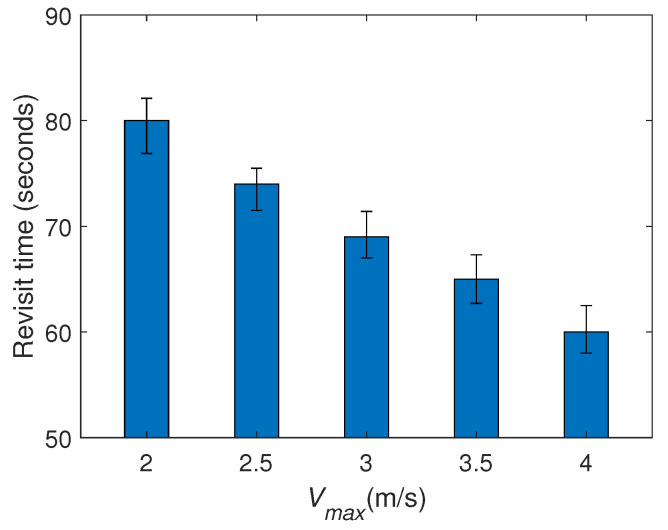
The impact of $V_{max}$ on the target revisit time.

Finally, we study the impact of a key parameter on the target revisit time: $V_{max}$, and we consider the maximum revisit time among all the targets. We set $V_{max}$ with various values and conduct more computer simulations on the last case. Specifically, the movements of the targets maintain the same in all the simulations. From Figure 12 we can see that increase the UAVs’ maximum linear speed significantly decreases. The reason is clear: the faster the UAVs move, the less time spent on the movements between targets. The maximum angular velocity $U_{max}$ also influences the target revisit time. But its impact is quite slight, because increasing $U_{max}$ only influences the movement for changing the motion direction, while it does not affect the time the UAVs spend on the movements between targets.

In summary, in this section, we have tested the proposed algorithms via computer simulations. The results show that the proposed online navigation algorithms can periodically survey a set of moving targets in a region. The region is not necessarily flat but can be uneven. The UAV maximum linear speed significantly affects the target revisit time while the maximum angular speed has a slight impact.

## 6. Conclusions

In this paper, we focused on the application of sensing coverage of moving targets by a UAV team. We presented an online path planning method for a single UAV to periodically cover a set of moving targets on the flat ground or the uneven terrain. The low-level control accounting for the dynamics of the UAV was considered in the path planning method. We further extended this method to navigate multiple UAVs for the sensing coverage of moving targets based on the shared information about targets among UAVs. Via computer simulations, we observed that the extended method may guide the UAVs to traverse the major area of the considered region, which may result in long revisit time. To avoid this, we adopted the Voronoi partition technique to narrow the movement range of each UAV. Simulation results showed that this improved method significantly reduces the revisit times of the moving targets.

One limitation of the current navigation method is based on the assumption that the targets move on some given trajectories. When these trajectories are not known to the UAVs, the target location predictions are with more uncertainty. As a result, the UAVs may lose some targets during the mission. Our future research work is to extend the proposed method to deal with this situation by including the searching operation when any target is lost.

## Figures and Tables

**Table 1:** The main notations used throughout the paper and their meanings.

Notation	Meaning
$p_{i}\left(t\right)$	Position of UAV *i* at time *t*
$h_{i}\left(t\right)$	Heading of UAV *i* at time *t*
$v_{i}\left(t\right)$	Linear speed of UAV *i* at time *t*
$u_{i}\left(t\right)$	Angular velocity of UAV *i* at time *t*
α	Observation angle
*Z*	Flight altitude
*R*	Radius of the vision cone of UAVs
$q_{i}\left(t\right)$	Position of target *j* at time *t*
${\hat{q}}_{i}\left(t\right)$	Estimated position of target *j* at time *t*
δ	Sampling time
ϵ	Maximum error of the estimated target speed
$\tau_{j}$	Revisit time of target *j*
$\beta_{j}\left(t\right)$	Uncertainty level of target *j* at time *t*
$\gamma_{j}\left(t\right)$	Weighted uncertainty level of target *j* at time *t*
$d_{ij}\left(t\right)$	Distance between UAV *i* and target *j* at time *t*
